# Early Detection of Jaw Malignancies: Insights From Three Clinical Case Reports for Dental Professionals

**DOI:** 10.7759/cureus.84174

**Published:** 2025-05-15

**Authors:** Oumayma El Yacoubi, Bouchra Taleb, Saloua Dghoughi

**Affiliations:** 1 Oral Surgery, Université Mohammed V de Rabat, Rabat, MAR

**Keywords:** detection, diagnosis, jaw, malignancies, malignant neoplasm

## Abstract

Malignant lesions of the jaw represent a heterogeneous group of pathologies that pose considerable diagnostic challenges for dental and maxillofacial practitioners. Early detection is essential, as these lesions often present with nonspecific symptoms and radiographic features that can mimic benign conditions or odontogenic infections, potentially leading to diagnostic delays.

This report presents three clinical cases of jaw malignancies, emphasizing their characteristic clinical presentations and radiological findings. The cases highlight the critical need for vigilance and a high index of suspicion when evaluating atypical intraoral symptoms. Prompt and accurate diagnosis of these neoplasms is fundamental to improving treatment outcomes and reducing associated morbidity and mortality.

## Introduction

Malignant neoplasms comprise a diverse array of lesions characterized by uncontrolled growth, the ability to invade adjacent tissues, and the potential for metastasis to distant sites. In the jaw, malignant neoplasms can be categorized into odontogenic and non-odontogenic lesions. These lesions are further classified as primary, which originate directly from the jaw’s tissues, or secondary, which arise from tumors originating in other anatomical sites. A lack of public awareness regarding the signs, symptoms, and risk factors, coupled with insufficient knowledge for early detection among healthcare providers, is believed to contribute to the diagnostic delays in identifying these lesions [1,2].

This article aims to present three cases of malignancies affecting the jawbones, offering detailed accounts of their clinical presentations and the corresponding radiographic findings.

## Case presentation

Case 1

A 12-year-old male patient was referred to the Outpatient Department of Surgery due to a painful swelling in the right mandible, initially diagnosed as cellulitis. Despite antibiotic management, the patient's condition showed no improvement. The patient had no significant medical history.

**Figure 1:**
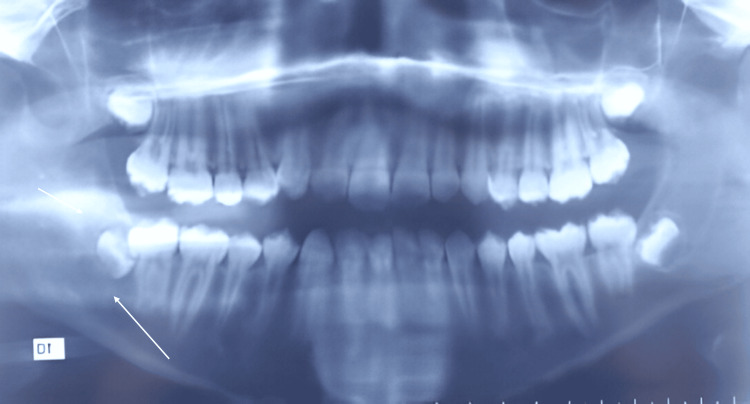
Orthopantomography shows a poorly defined radiolucent image adjacent to the right mandibular third molar (arrow)

A clinical examination revealed facial asymmetry due to a sensitive, firm swelling on the right mandibular angle, with no associated cervicofacial lymphadenopathy. Intraoral evaluation detected a significant swelling on the internal aspect of the ascending ramus. Panoramic radiography showed a poorly defined radiolucent image adjacent to the right mandibular third molar (Figure 1).

**Figure 2:**
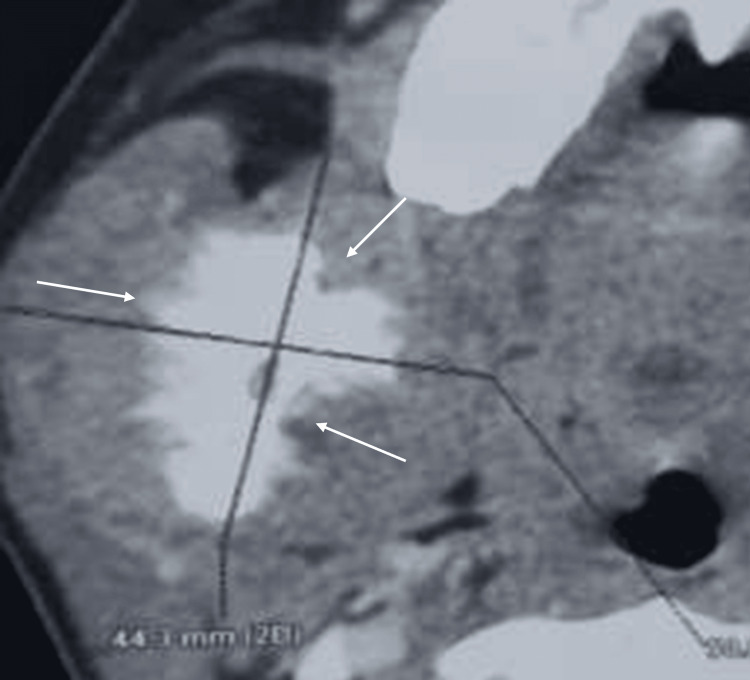
Axial view of the CT scan with soft tissue window illustrates hypodensity accompanied by a sunburst periosteal reaction affecting both the internal and external cortical surfaces of the ramus (arrows) CT, computed tomography

An axial view from a computed tomography (CT) scan utilizing a bone window setting illustrated a sunburst periosteal reaction affecting the internal and external cortices of the ramus (Figure 2). The parenchymal window revealed a mass measuring approximately 5 cm.

**Figure 3:**
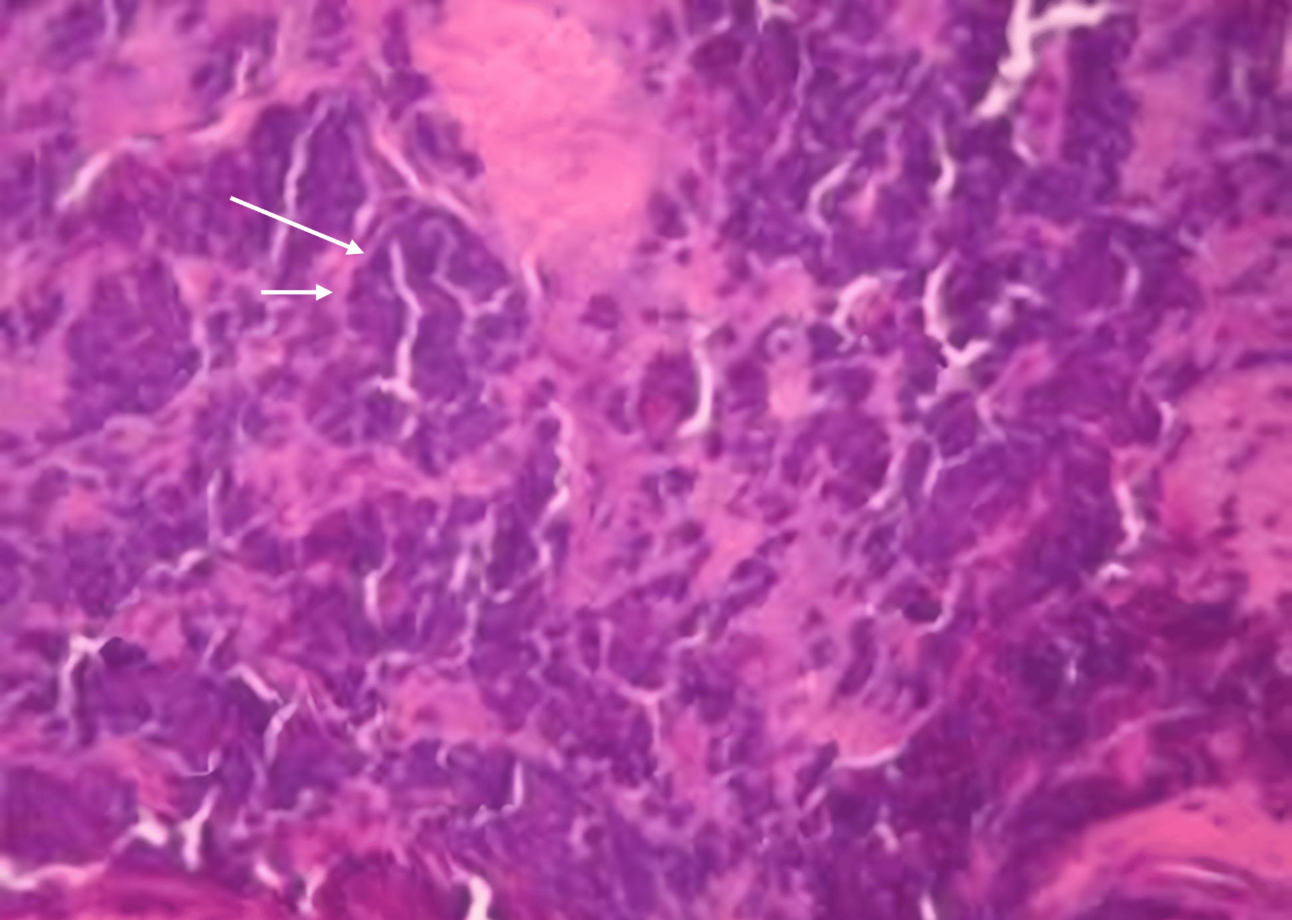
Small, round tumor cells with a low abundance of cytoplasm, hyperchromatic and mitotic oval nuclei (arrows; hematoxylin and eosin (H&E) staining, magnification ×100)

Considering the clinical presentation and radiological findings, a malignant tumour was provisionally diagnosed. An incisional biopsy was performed under local anesthesia, and the specimen was submitted for histopathological analysis, which confirmed the diagnosis of Ewing's sarcoma (Figure 3).

The patient was subsequently referred to an oncology center for appropriate treatment (radiotherapy and chemotherapy). Follow-up evaluations revealed significant improvement in both clinical and radiological findings, and the patient is now in a state of clinical remission.

Case 2

A 40-year-old female patient, with no significant history of systemic disease, was presented to the Oral Surgery Department with a right mandibular swelling that had progressed over the past four months. The patient reported a history of removal of the first mandibular molar 10 months prior to the onset of the swelling, which was initially considered to be an abscess. The patient received multiple courses of antibiotics, including amoxicillin, metronidazole, and spiramycin, for a duration of 15 days, without any improvement in symptoms.

An extraoral examination revealed a painless swelling in the right mandible, causing facial asymmetry. Additionally, fixed, painless right submandibular lymphadenopathy and hypoesthesia of the labiomental region were noted, with no associated motor disturbances or reflex abnormalities. Intraoral examination demonstrated a firm, buccal swelling that extended from the right mandibular premolar region to the molar area, reaching the inferior border of the mandible.

Pulp sensitivity tests indicated that the first and second premolars, as well as the third molar, were vital, suggesting no endodontic involvement at that stage, while the right mandibular second molar tested non-vital. Mobility level 2 (Miller’s classification) was observed in the second and third right mandibular molars.

**Figure 4:**
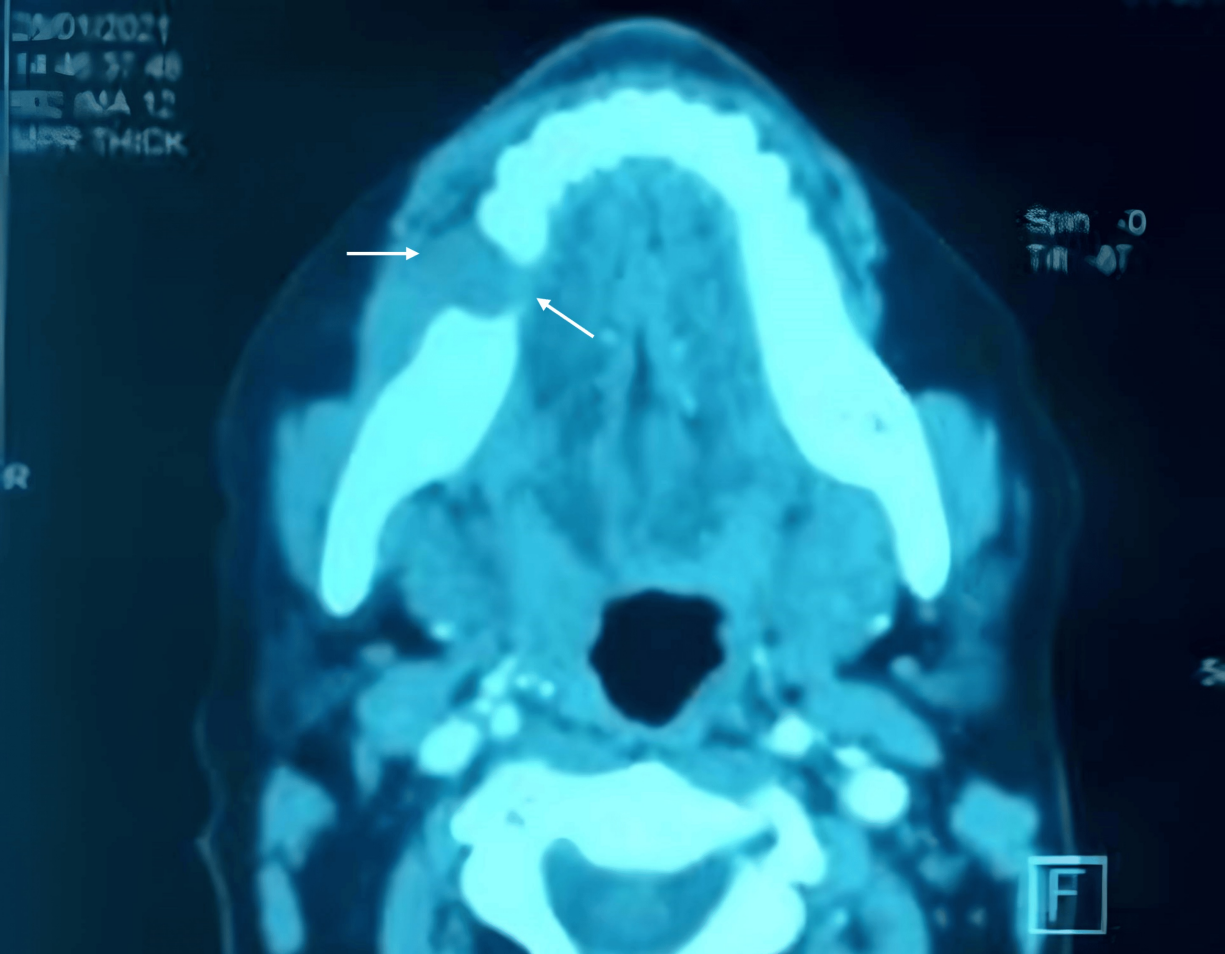
Radiographic presentation showing a cross-sectional view from a CT scan with a soft tissue window revealed a poorly defined hypodense image at the site of the right mandibular first molar (46; arrows) CT, computed tomography

A cross-sectional view from a CT scan with a bone window revealed a poorly defined hypodense image at the site of the right first mandibular molar, which limited the inferior alveolar canal and demonstrated continuity with the apical region of the right second molar. Additionally, a well-defined, hyperdense, rounded image of small dimensions was noted in the apical region of the right second molar. In the soft tissue window, a mass with muscular density was identified, arising from the extraction site (Figure 4).

Based on the clinical and radiological findings, differential diagnoses included central giant cell granuloma, myxoma, ameloblastoma, and malignant tumors.

**Figure 5:**
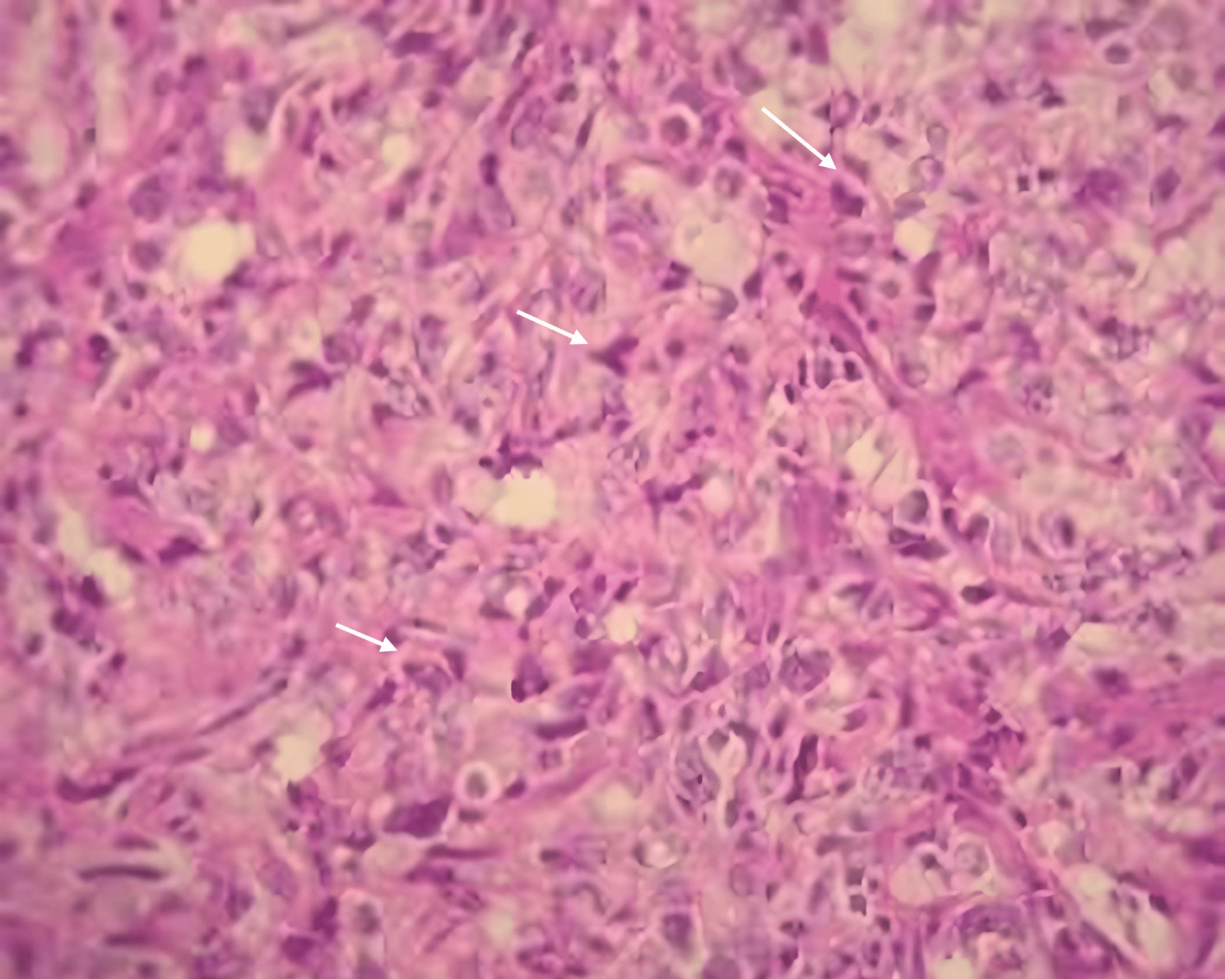
Diffuse, dense circular cellular proliferation (arrows; hematoxylin and eosin (H&E) staining, magnification ×400)

An incisional biopsy was performed on the affected area, followed by histopathological and immunohistochemical analysis. The biopsy results confirmed a diagnosis of small-cell non-Hodgkin lymphoma (NHL) (Figure 5). The patient was promptly referred to an oncologist to initiate appropriate treatment (chemotherapy).

Case 3

A 25-year-old male patient, with no significant medical history, presented with a vestibular swelling in the left anterior maxilla that had progressed over the past four years. According to the patient, this swelling developed following the extraction of the temporary left maxillary canine and root canal treatment of the left lateral incisor. The patient had no history of tobacco or alcohol use.

Extraoral examination revealed no abnormalities. Intraoral assessment showed a fluctuating swelling approximately 2 cm in size, affecting the vestibular and palatal alveolar processes near the left upper canine and first premolar. The lesion presented with an ulceration, raised border edges, and was tender and bleeding on contact.

**Figure 6:**
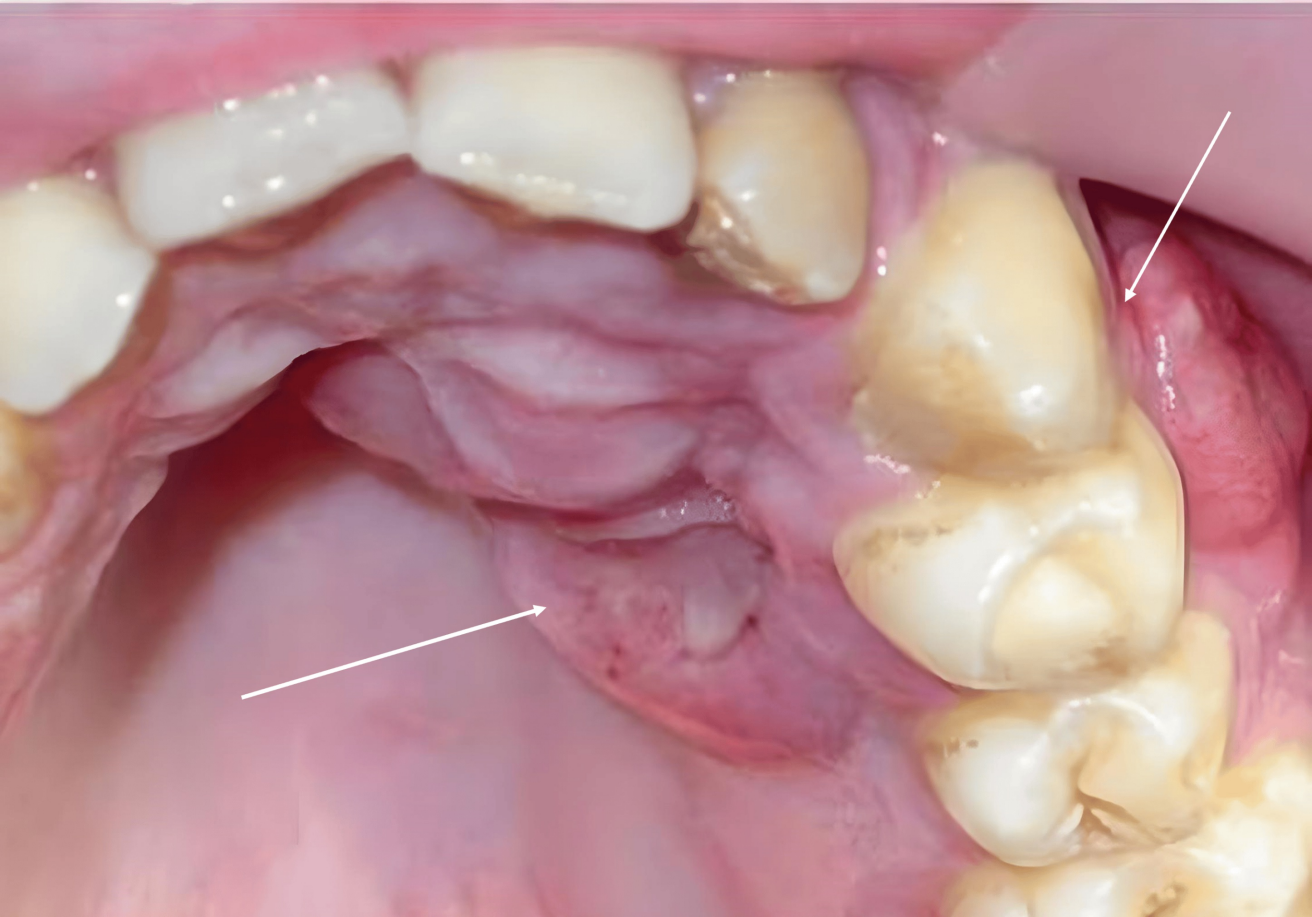
Intraoral view exhibits swelling affecting the vestibular and palatal alveolar processes near the left upper canine and first premolar (arrows)

A vestibular fistula was observed, discharging pus from the attached gingiva adjacent to the left canine. The canine and first premolar exhibited increased mobility but maintained a positive vitality response (Figure 6)*.*

**Figure 7:**
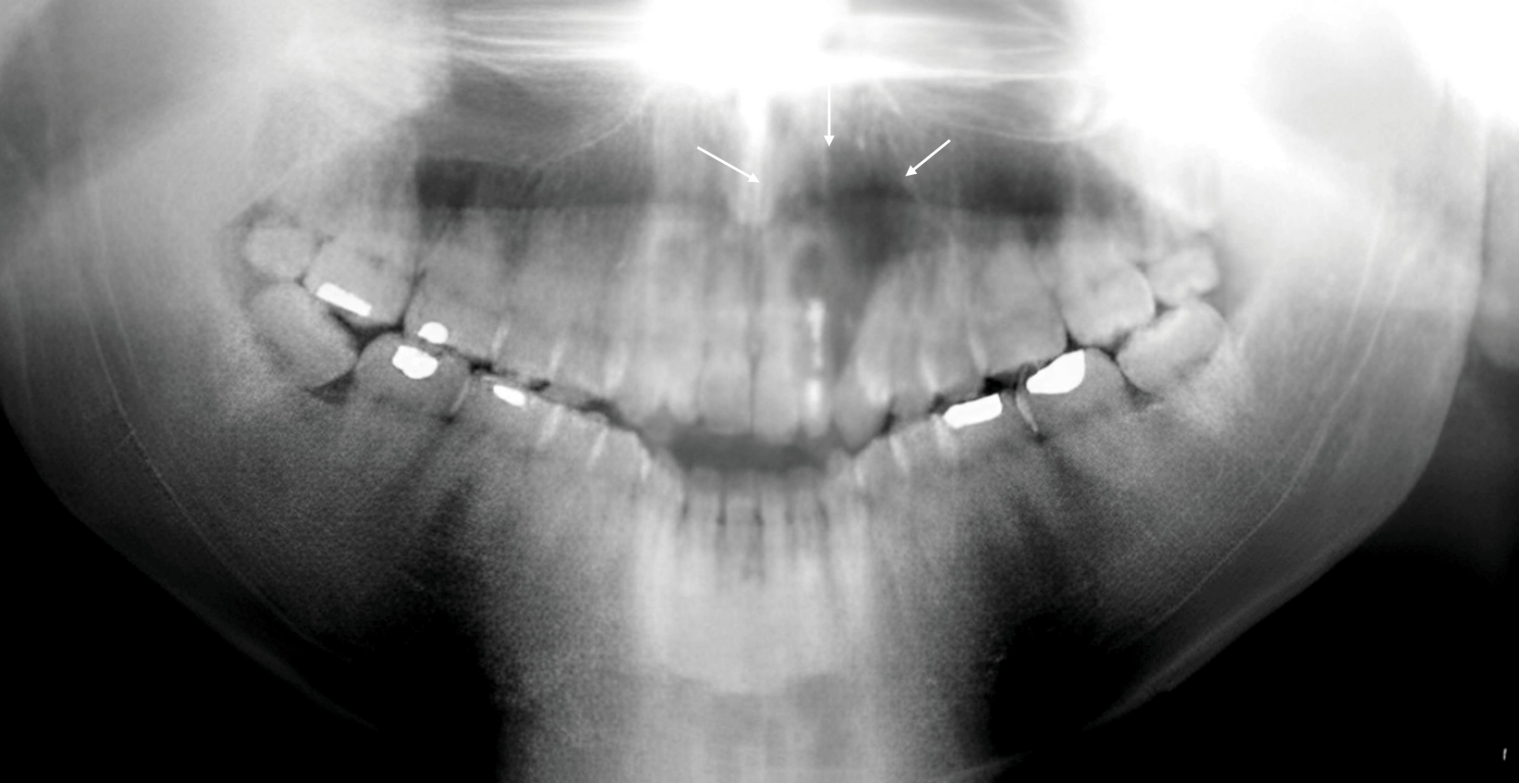
Panoramic radiograph shows a well-defined "inverted pear-shaped" radiolucency, extending from the central incisor to the left first premolar (arrows)

The panoramic radiograph demonstrated a well-defined "inverted pear-shaped" radiolucency measuring 2 x 3 cm, extending from the central incisor to the first premolar. Defective endodontic treatment was evident on the left lateral incisor, along with root resorption (Figure 7).

Based on the clinical presentation and radiographic findings, a radicular cyst was suspected due to the slowly progressive swelling and inadequate endodontic treatment associated with the upper left lateral incisor. Consequently, endodontic treatment was resumed, and follow-up was instituted.

Three months later, the patient presented with bilateral fixed submandibular and jugular lymphadenopathy, measuring approximately 4 cm. Intraoral examination revealed two vestibular ulcerations on the alveolar mucosa (buccal and palatal) in the area of the left upper canine and lateral incisor. Mobility level 2 was noted in the left lateral incisor, canine, and first and second premolars. Axial and coronal views from a CT scan revealed extensive bony destruction with ill-defined margins, extending to the area of the second premolar. No invasion of the maxillary sinus was observed.

**Figure 8:**
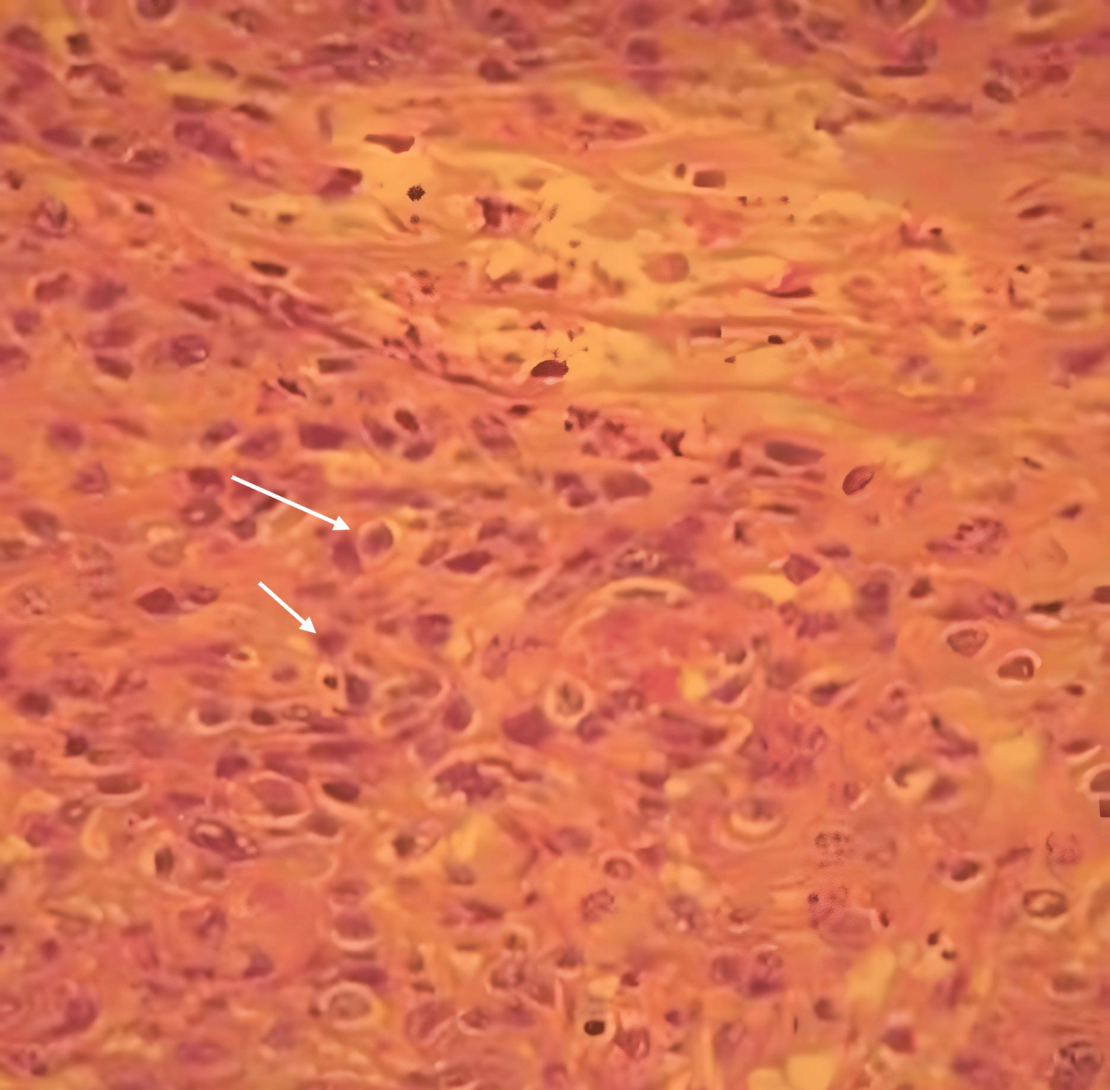
Microscopic images confirming the diagnosis of a keratinizing, moderately differentiated squamous cell carcinoma (arrows; hematoxylin and eosin (H&E) staining, magnification ×400)

These findings - including ulceration, tooth mobility, lymphadenopathy, and ill-defined radiolucency - raised suspicion of a malignant process. The patient was subsequently referred to the Maxillofacial Surgery Department, where a biopsy was performed, revealing primary intraosseous squamous cell carcinoma upon histopathological examination (Figure 8). Appropriate treatment was then initiated (surgery and radiotherapy).

## Discussion

Malignancies affecting the jawbone represent a wide range of neoplasms that necessitate specific management strategies and present distinct prognostic implications [1,2].

Malignancies that can involve the jawbone include primary tumors, which develop directly within the maxilla or mandible, as well as tumors originating from neighboring soft tissues that invade the bone structures - a process known as local bone infiltration. Additionally, malignant lesions may occur due to dissemination from the hematopoietic or lymphatic systems. Another category comprises metastatic tumors, which are secondary lesions resulting from malignancies that originate at distant sites and spread to the jawbone [1-3]. Malignant tumors of the jaws are categorized into two principal groups: odontogenic tumors, which originate from the embryonic tissues associated with tooth development, and non-odontogenic tumors, which encompass neoplasms of epithelial origin (carcinomas), mesenchymal origin (sarcomas), and hematopoietic origin (lymphomas) [3,4].

According to Global Cancer Observatory data, malignant tumors of the oral cavity have a prevalence of 4.69 per 100,000 in Morocco over the past five years, across all age groups. Primary tumors are more frequently observed in the jawbones than metastatic tumors, and non-odontogenic tumors are more common than odontogenic tumors.

The male-to-female sex ratio is 2:1, and the age of onset of tumors varies significantly according to their histological type. Squamous cell carcinoma, which accounts for 90% of malignant tumors of the oral cavity, is typically diagnosed after the age of 40. In contrast, certain conditions, such as Ewing's sarcoma and Hodgkin's lymphoma, tend to manifest at a younger age [5,6].

Diagnostic approach

The oral cavity presents significant accessibility for clinical evaluations, facilitating the early detection of malignant neoplasms by proficient clinicians. Early diagnosis is crucial, as it substantially influences patient prognosis, ultimately reducing morbidity and improving the overall quality of life [7,8]. The diagnostic approach for suspected malignant tumors of the jaw typically encompasses the following steps.

Patient History

The first step in the screening of oral cancer is to attain a detailed and comprehensive patient history that includes medical history with a list of current medications; dental history, including past dental visits and treatments performed; and history of oral habits and lifestyle, with specific reference to frequency (tobacco, alcohol, etc.).

The following elements can be collected during the anamnesis and may lead to suspicion of malignancy: general signs of malignancy in the jaw area include systemic symptoms such as weight loss, fatigue, night sweats, and loss of appetite [9,10]. It is also crucial to consider the patient's oncological history, particularly the possibility of metastases, which is especially concerning in cases of primary cancers like breast, kidney, lung, and colon cancer, that may spread to the jaw. Moreover, pre-existing conditions that predispose individuals to malignant tumor development should be taken into account, such as underlying bone disorders like fibrous dysplasia [10,11].

Clinical evaluation

Extraoral Signs

Lymphadenopathy: Certain characteristics of lymphadenopathies may suggest malignancy. Tenderness upon palpation is generally associated with inflammatory diseases. In contrast, firm and fixed lymphadenopathies, which are fixed to adjacent structures, strongly suggest a malignant origin. In the three cases reported, the patients exhibited firm, fixed, and painless lymphadenopathy.

Generalized polyadenopathy is more commonly associated with systemic conditions or hematologic cancers, such as acute lymphoblastic leukemia, NHL, and Hodgkin disease. Conversely, lymphadenopathies associated with solid tumors are typically localized to a single region, corresponding to the drainage territory of the affected lymph node [10,11].

Cancer-related neuropathy may be caused by direct tumor invasion or compression of nerve structures. The clinical manifestations of cancer-related neuropathy are indistinguishable from those of other distal peripheral neuropathies, as in our second case.

Patients may initially present with hypoesthesia, paresthesia, or dysesthesia, which may occasionally be the first presenting symptoms of an underlying malignancy.

Specific neurological syndromes that may suggest a malignancy diagnosis have been identified. For example, a lesion of the submandibular branch of the trigeminal nerve, colloquially referred to as "numb chin syndrome," is strongly associated with underlying malignancy and metastatic disease. It precedes a diagnosis of malignancy in up to 47% of cases, and it’s most commonly associated with metastatic breast cancer and lymphoma, followed by prostate cancer, lung cancer, and leukemia [12].

Patients typically present with anesthesia of the lower lip or chin, often unilaterally, though bilateral involvement can occur. In the absence of an alternative known etiology, such a presentation should prompt a thorough workup for metastatic disease [12,13].

Swelling: A rapid decrease in mouth opening (generally reflecting muscular invasion), coupled with fast-growing, painful swelling that does not show signs of infection and does not improve with medical treatment, serves as a warning sign that the lesion is likely malignant [10].

Intraoral Signs

Swelling, lack of healing at the extraction site, and tooth mobility without evidence of periodontal disease are the main indicators of malignancy in the oral cavity. The covering mucosa may show ulcerative-vegetative or hemorrhagic lesions with raised borders. Nevertheless, in cases of non-externalized primary jaw lesions, the mucosa may appear normal. Other mucosal signs may include pallor, gingival hemorrhage, petechiae, and gingival hypertrophy, which could indicate hematopoietic malignancy [1-14]. 

Radiographic evaluation

Radiography plays a crucial role in complementing the clinical examination, enabling the identification of suspicious radiographic signs and assessing their extent to guide therapeutic decisions.

Conventional Radiography

The orthopantomogram serves as the first-line examination, as it provides a comprehensive evaluation of the dentomaxillary structures while maintaining an optimal cost-to-radiation ratio [14]. 

Signs suggesting malignancy in conventional imaging techniques include irregular widening of the periodontal ligament space (PLS), destruction of the lamina dura, spiking resorption of roots, extensive bone destruction, and an irregular radiolucent image that is poorly defined. Additionally, delayed healing of extraction sockets, loss of mandibular canal cortication, and the appearance of floating teeth are also indicative of possible malignancy.

3D Imaging

Primarily, CT with bone and soft tissue windows provides a detailed 3D analysis of the bony structures, allowing for a precise assessment of tumor location and extent [5]. Signs suggesting malignancy on 3D imaging include the destruction of cortical borders, multifocal and ragged radiolucent foci or rarefaction in trabecular bone, as well as sunray or spiculated periosteal reactions. The presence of a Codman triangle periosteal reaction, widening of neural foramina, and a mass invading adjacent structures, such as the sinus or nasal cavities, are also significant indicators. Unlike benign lesions, which tend to displace neighboring structures, malignant lesions often invade them [14].

Other complementary tests

Biological exams may be indicated, particularly a complete blood count (CBC), which can be prescribed in cases of suspected leukemia or other hematological malignancies. Biopsy remains the gold standard, as it provides a tissue sample for histopathological analysis, which is essential for establishing a definitive diagnosis. Afterward, an extension workup is recommended, allowing for cancer staging according to the TNM (Tumor, Node, Metastasis) classification and helping to tailor the therapeutic treatment accordingly [1].

Differential diagnosis

Early-stage malignant lesions can exhibit a clinical and radiographic presentation that is nonspecific and similar to odontogenic infections and various benign neoplasms of the oral cavity, as exemplified by our three cases [1].

In the first and second cases, the painful nature of the swelling may lead to a misdiagnosis of odontogenic infection, such as cellulitis. This emphasizes the importance of a thorough clinical examination to rule out a dental infection as the cause. Furthermore, the interpretation of radiographic signs, such as a diffuse and ill-defined radiolucent image and a periosteal reaction in a sunburst pattern, is crucial for refining the diagnosis.

In the last case, we initially considered the lesion to be a periapical cyst due to its radiographic presentation and association with a tooth presenting inadequate endodontic treatment. However, the morphological changes of the lesion during endodontic therapy, along with the development of regional adenopathy, provided additional support for the malignancy diagnosis (Table 1) [14].

**Table 1:** Summary of clinical and radiographic findings in three cases

Case	Initial Diagnosis	Clinical Features	Radiographic Findings	Revised Diagnosis	Reason for Revision
First case	Odontogenic infection (cellulitis)	Painful swelling	Diffuse, ill-defined radiolucency; periosteal sunburst pattern	Ewing's sarcoma	Poorly defined radiolucent image
Second case	Odontogenic infection (cellulitis)	Painful swelling	Diffuse, ill-defined radiolucency; periosteal sunburst pattern	Small-cell non-Hodgkin lymphoma	Fixed painless submandibular right lymphadenopathy and hypoesthesia of the right lip
Third case	Periapical cyst	Swelling	Well-defined radiolucency and poor endodontic treatment	Primary intraosseous squamous cell carcinoma	Morphological changes and regional adenopathy during therapy

## Conclusions

Malignant lesions of the jaw represent a diverse group of pathologies that pose significant diagnostic challenges for dental and maxillofacial professionals. Recognizing the signs of malignancy early is essential, as prompt diagnosis and treatment can help reduce associated mortality and morbidity.

In summary, a thorough clinical examination is essential for detecting early signs of malignancy. If initial treatments are ineffective, further investigations - including advanced imaging and biopsy - are crucial.
